# Extracellular Vesicles After Allogeneic Hematopoietic Cell Transplantation: Emerging Role in Post-Transplant Complications

**DOI:** 10.3389/fimmu.2020.00422

**Published:** 2020-03-20

**Authors:** Giuseppe Lia, Clara Di Vito, Marco Cerrano, Lucia Brunello, Francesca Calcaterra, Marta Tapparo, Luisa Giaccone, Domenico Mavilio, Benedetto Bruno

**Affiliations:** ^1^Stem Cell Transplant Program, Department of Oncology, A.O.U. Città della Salute e della Scienza di Torino, Turin, Italy; ^2^Department of Molecular Biotechnology and Health Sciences, University of Turin, Turin, Italy; ^3^Unit of Clinical and Experimental Immunology, Humanitas Clinical and Research Center, Milan, Italy; ^4^Department of Medical Biotechnologies and Translational Medicine (BioMeTra), University of Milan, Milan, Italy; ^5^Department of Medical Sciences, Molecular Biotechnology Center, University of Turin, Turin, Italy

**Keywords:** extracellular vesicles, allo-HCT, immune-reconstitution, GvHD, disease-relapse

## Abstract

Extracellular vesicles (EVs) play an important role in the cellular crosstalk by transferring bioactive molecules through biological barriers from a cell to another, thus influencing recipient cell functions and phenotype. Therefore, EVs are increasingly being explored as biomarkers of disease progression or response to therapy and as potential therapeutic agents in different contexts including in hematological malignancies. Recently, an EV role has emerged in allogeneic hematopoietic cell transplantation (allo-HCT) as well. Allogeneic hematopoietic cell transplantation often represents the only curative option in several hematological disorders, but it is associated with potentially life-threatening complications that can have a significant impact on clinical outcomes. The most common complications have been well-established and include graft-versus-host disease and infections. Furthermore, relapse remains an important cause of treatment failure. The aim of this review is to summarize the current knowledge, the potential applications, and clinical relevance of EVs in allo-HCT. Herein, we will mainly focus on the immune-modulating properties of EVs, in particular those derived from mesenchymal stromal cells, as potential therapeutic strategy to improve allo-HCT outcome. Moreover, we will briefly describe the main findings on EVs as biomarkers to monitor graft-versus-host disease onset and tumor relapse.

## Introduction

Allogeneic hematopoietic cell transplantation (allo-HCT) is an effective therapeutic procedure applied to a broad range of hematological disorders, most frequently acute leukemias and myelodysplastic syndromes (1). Hematopoietic cell transplantation consists of the intravenous infusion of hematopoietic stem and progenitor cells, from a fully or partially human leukocyte antigen (HLA)–matched healthy donor, which aims to reestablish a normal hematopoiesis and immune functions. Before HCT infusion, a conditioning regimen is necessary to provide an empty stem cell niche in the host bone marrow (BM) for new stem cells. Following engraftment, allo-HCT contributes to control the underlying malignancy through a graft-versus-leukemia (GvL) effect that is mainly mediated by donor-derived alloreactive T cells and/or natural killer (NK) cells (2). However, HCT is still limited by potentially life-threatening complications, the management of which has markedly improved, although still associated with high morbidity and mortality (3).

The most important complications after allografting are acute and chronic graft-versus-host disease (GvHD), which remain the main cause of morbidity and mortality despite the high number of clinical trials aimed at improving prophylaxis and therapy (4, 5).

Acute GvHD (aGvHD) usually develops within 100 days after allo-HCT in 30% to 50% of patients (1). Typical aGvHD target organs are the skin, gastrointestinal tract, and liver. Chronic GvHD (cGvHD) is a pleiotropic entity observed in 30–70% of patients and deeply affects patients' quality of life. It involves potentially most organ systems including, among the others, the lung, oral mucosa, eyes, joints, hair and nails, musculoskeletal, and genital tract (6, 7).

Graft-versus-host disease occurs when immune cells of donor origin recognize the recipient tissues as foreign. The first step in aGvHD pathogenesis is the conditioning regimen-induced tissue damage and infiltration of the gastrointestinal tract by neutrophils and monocytes. Moreover, release of reactive oxygen species, DAMP (damage-associated molecular pattern), and PAMP (pathogen-associated molecular pattern) molecules elicit inflammation and activation of both innate and adaptive immune responses (8). Donor alloreactive T cells recognizing major or minor histocompatibility antigens of the host is the key event in aGvHD pathogenesis. The targeting of host cell death is mediated by the expression of Fas Ligand and by release of granzyme B and perforins (9). Another significant factor in aGvHD pathogenesis is the production of cytokines and chemokines [e.g., interleukin (IL)-1, interferon γ (IFN-γ), tumor necrosis factor (TNF), IL-6] that can directly and indirectly exert cytotoxicity (10).

Chronic GvHD pathogenesis consists of three phases: the first phase is characterized by tissue damage and production of DAMPs and PAMPs as in aGvHD, resulting in activation of antigen-presenting cells (APCs) and T cells. During phase 2, priming and expansion in lymph nodes and thymus of B lymphocytes and T cells (mostly T helper (TH) 1, 2, and 17), respectively, are observed. Of note, thymus injuries due to the conditioning regimens have been associated with reduced generation of regulatory T cells. Then, deposition of extracellular matrix and fibrosis (third phase) is the result of chronic inflammation and fibroblast activation (11). Immunosuppressive agents are needed to prevent and treat GvHD.

Following HCT, a prolonged state of immunodeficiency is observed (12). Therefore, patients are exposed to the risk of infectious complications, often severe and difficult to treat.

Unfortunately, the immunosuppressive agents can also reduce the beneficial GvL effects, leading to an increased risk of disease relapse. Indeed, disease relapse still represents the major cause of allo-HCT failure, and many efforts are being made to prevent it, including immunosuppression modulation, disease-specific drug intervention, or delayed lymphocyte infusions, which can be used alone or in combination (13). In this context, early detection of disease reappearance is particularly important (14), because results are commonly dismal after an overt relapse has occurred (15).

In this review, we will discuss the main characteristics of extracellular vesicles (EVs), which make them very attractive for the development of their potential application as biomarkers for the most common post–allo-HCT complications or EV-based therapeutic strategy. Furthermore, we will focus on the immune-modulating properties of EVs derived from mesenchymal stromal cells (MSCs), which have been widely characterized in allo-HCT field.

## Extracellular Vesicles

**Figure 1:**
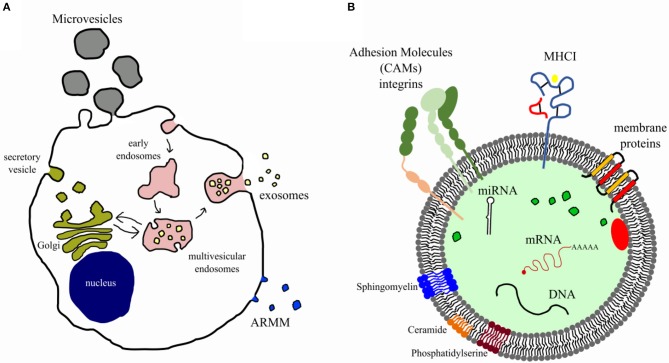
Biogenesis and composition of extracellular vesicles. **(A)** Diagram illustrates the well-accepted model for extracellular vesicle biogenesis. Microvesicles and ARMM [arrestin domain–containing protein 1 (ARRRDC1)–mediated microvesicles] originate from budding of plasma membrane, whereas exosomes from the endosomal compartment (multivesicular endosome). **(B)** EVs carry several bioactive molecules such as membrane and intraluminal proteins (e.g., adhesion molecules, MHCI), lipids (e.g., lipid raft, sphingomyelin, disaturated lipids, phosphatidylserine, ceramide), nucleic acids (miRNAs, genomic and mitochondrial DNA, and mRNA), and organelles.

Extracellular vesicles are membrane enclosed particles, secreted by virtually all cell types and containing different biomolecules, including nucleic acids, proteins, lipids, and carbohydrates (16). In recent years, several studies demonstrated that EVs play an essential role in intercellular communications, thus being involved in regulation of physiological homeostasis, as well as in pathological states by influencing cell proliferation, differentiation, organ homing, injury and recovery, and disease progression (17). Extracellular vesicles can be further classified based on their dimension and origin (16). The term “extracellular vesicles” is widely used mainly to describe the two most abundant EV populations, that are the microvesicles (MVs), which originate from outward protrusion or budding of the plasma membrane, and the exosomes (EXs) of endosomal origin (Figure 1).

**Figure 2:**
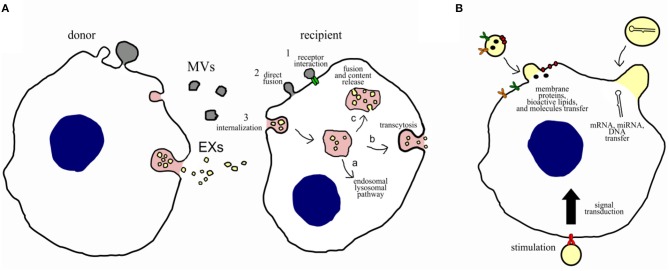
Potential communication mechanisms and biological functions of EVs. **(A)** Potential intercellular communication mechanisms between donor cells and recipient cells. Intercellular communication can occur: (1) direct interaction of ligands expressed on the surface of EVs with receptors on the recipient cell membrane; (2) direct fusion of the EVs with the cell membrane of the recipient cell, resulting in the release of their content; or (3) internalization through the endocytic pathway, which can result in (a) degradation via the lysosomal pathway, (b) transcytosis, or (c) fusion of the EVs with membrane of the endosome, resulting in content release. **(B)** Potential biological functions of EVs on recipient cells. Microvesicles and exosomes may dock at the plasma membrane of a target cell. Bound vesicles may either fuse directly with the plasma membrane or be endocytosed. Both pathways result in the delivery of proteins, lipids, and RNAs into the membrane or cytosol of the target cell. Binding of EVs to specific receptors can stimulate recipient cells through a signal transduction or by transferring receptors into the recipient membrane.

Extracellular vesicles target recipient cells by surface molecules, and once attached, they can induce intercellular signaling via receptor–ligand interaction (Figure 2); alternatively, they can be internalized by endocytosis and/or phagocytosis, or they can fuse directly with the plasma membrane releasing their cargo (miRNAs, proteins, and other bioactive molecules) (18). The cargo content could have short- and long-term implications on target cell phenotype and function. For example, miRNAs could negatively regulate complementary mRNA, after being released, mediating its cleavage with subsequent degradation or translation inhibition.

Because different cell types can release discrete subpopulations of EVs, each with different proteomic and RNA cargo and membrane protein composition, they can mediate different biological and sometimes opposite effects, because of their vast heterogeneity and specificity (19–23).

**Figure 3:**
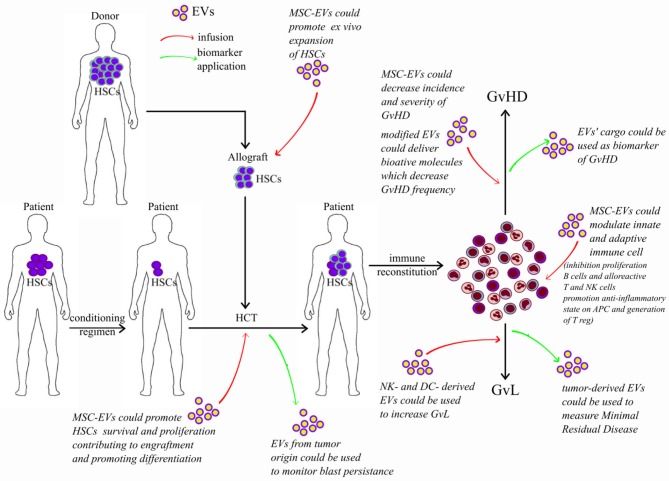
Potential application of extracellular vesicles in allografting. Red arrows represent potential effect of infused EVs; green arrows represent potential application of EVs as biomarkers. HSC, hematopoietic stem cells; HCT, hematopoietic cell transplantation; EVs, extracellular vesicles; MSCs, mesenchymal stromal cells; GvL, graft-versus-leukemia; GvHD, graft-versus-host disease.

Because of the therapeutic potential of EVs and to better understand their pathophysiological role, many studies have been designed to identify in EVs molecules responsible of their great effect and to serve as biomarkers. In this context, it has been observed that EVs released from immune or regulatory cells can partially regulate immune responses. This property has great therapeutic potential in allo-HCT, in which immune cells play a major role in mediating GvL effects and reducing GvHD (Figure 3).

### EV Production and Characterization

The importance of the starting material (cell cultures, tissue specimen, biological fluids) and its preprocessing (time of harvest, storage) are considered crucial for EV applications. Recently, the International Society of Extracellular Vesicles (ISEV) established general guidelines to uniform EV collection and characterization (24). Some experiments were conducted to asses EV stability in plasma and serum under different storage conditions and concluded that storage temperature does not significantly affect EV stability as well as their cargo (25). Conversely, the presence of different contaminants (such as lipoproteins, protein complexes, platelets), patient-related variables (age, gender, time of collection, etc.), and source of EVs should be carefully considered (24).

Another critical point is the isolation method. As a matter of fact, many techniques developed in recent years are more suitable for research rather than clinical applications. At present, the gold standard protocol is the differential ultracentrifugation (24), which could be coupled with other techniques such as density gradients, precipitation, filtration, size exclusion chromatography (SEC), and immune isolation to eliminate contaminants (24). However, this method would be difficult to translate into the clinical setting, given its high cost and lack of automatization (26).

The potential EV application in clinical practice requires user-friendly, cheaper, and faster methods for EV isolation and characterization. Moreover, the introduction of EVs as therapeutic agent needs methods that allow high yield and purity. Tangential-flow fractionation and SEC meet those requirements with minimal manipulation of the starting material. Tangential-flow fractionation separates particles in a filter column containing hollow fibers applying a tangential flow. Size exclusion chromatography isolates EVs according to their size, relying on the correlation between elution volume or diffusion coefficient and the molecule hydrodynamic radius. Both methods could be coupled to obtain a scalable and Good Manufacturing Practices grade product (27–29).

Extracellular vesicle application as biomarkers does not necessarily require big yields and purity collection methods. Commonly used techniques with a high translational potential are precipitation-based protocols that allow fast and user-friendly EV isolation for further biomarker identification analyses. In addition, direct immunoaffinity capture, which employs immunomagnetic beads to isolate and characterize EVs, is a suitable technique easy to apply in the clinical setting. This technique allows the concomitant isolation of specific subpopulation of EVs and in part their characterization (30).

New lab-on-chip methods have been proposed as diagnostic platforms (31) and can be coupled with high-throughput procedures offering the possibility to extend EV research into routine diagnostic and therapeutic settings.

Different methods can be used to characterize the concentration and size of EVs (32). Dynamic light scattering and nanoparticles tracking analysis rely on the Brownian motion of particles to measure size distribution and concentration of EVs. Both techniques are widely employed, although data might be influenced by EV composition and presence of contaminants, as lipoproteins (24). Others methods for size measurement that are recommended by ISEV are flow cytometry (33) and resistive pulse sensing (34).

Electron microscopy and atomic force microscopy are more precise tools that allow size and morphology evaluation of EV population (32). Unfortunately, these techniques do not allow further cargo characterization that should be investigated for clinical application. To identify the molecules responsible of EV biological activity, -omic approaches such as RNAseq, Raman spectroscopy, mass spectrometry, and lipidomic analyses are required (24, 32).

## Therapeutic Potential of EVs in allo-HCT

### MSC-Derived EVs and Modulation of the Immune Response

Mesenchymal stromal cells are fibroblast-like multipotent cells that can be isolated from different tissues, including BM, umbilical cord (UC), and adipose tissue (35). In the BM niche, these cells play an important role in controlling hematopoietic stem cell (HSC) fate (36). In detail, BM-MSCs support hematopoiesis expressing multiple adhesion molecules necessary for cell–cell and cell–matrix interactions, homing, and mobilization of HSCs (37).

It is widely assumed that the ability of MSCs to support hematopoiesis is also mediated by the constitutive secretion of several soluble factors, such as stem cell factor, leukemic inhibitory factor, and IL-6 (38–40), thus affecting HSC expansion and differentiation in a paracrine manner (41–44). Moreover, MSCs can be easily isolated from different human tissues, and they possess immune-modulatory properties, influencing both adaptive, and innate immune responses (45). For these reasons, allogeneic MSCs appear as a promising source for cell replacement strategies and have been tested for the treatment of several diseases, including acute injuries, such as ischemic stroke or myocardial infarction. However, in non-immunocompromised patients, allogeneic MSCs are rapidly rejected by the recipient immune system (46).

Growing evidence suggests that the paracrine effect of MSCs could be at least partially mediated by MSC-derived EVs (MSC-EVs). In this regard, by analyzing the miRNA and protein expression profile in MSCs and MSC-EVs both in normal and inflammatory conditions, Adamo et al. (47) observed the presence of several molecules such as MOES, LG3BP, PTX3, and S10A6 proteins; miR155; and miR497 involved in immunological processes. Different *in silico* approaches have also investigated the correlation between miRNA and protein expression profile and then evaluated the putative molecules or pathways involved in immunoregulatory properties of MSC-EVs.

Thus, given their possible involvement in hematopoiesis and immune homeostasis, MSC-EVs have been studied as an alternative therapeutic tool in a variety of preclinical models of immune disorders, including autoimmune diseases (48, 49) and GvHD in allo-HCT recipients (50–52).

#### MSC-EV Effect on Adaptive Immune Cells

Several lines of evidence demonstrated that MSC-EVs can influence adaptive immunity by modulating both T and B lymphocyte activity. Mesenchymal stromal cell–derived EVs are able to suppress T-cell proliferation and to promote a tolerogenic environment. Indeed, in an experimental murine model of autoimmune encephalomyelitis, it has been first observed that BM-MSC-MVs can act on T lymphocytes by inhibiting their proliferation and by promoting apoptosis of activated T lymphocytes and the generation of T regulatory cells (Tregs) (48). This evidence has been further confirmed in rodent models of allogeneic skin graft, liver injury, and islet transplantation using human EXs from embryonic- and BM-derived MSCs (53–55).

In agreement, human *in vitro* experiments on adipose-derived MSC-EXs demonstrated that EXs can inhibit the proliferation and differentiation of T cells as well as their IFN-γ production ability (56). Similarly, both EXs and MVs derived from BM- and UC-MSCs are able to suppress T-cell activation and to drive the expansion of Tregs in both healthy controls and type 1 diabetes patients (57–60).

This inhibitory effect of MSC-EVs on T-cell proliferation has been hypothesized to be mediated by the up-regulation of intracellular pathways, such as indoleamine 2,3-dioxygenase (IDO) (61, 62), despite no significant change in IDO activity has been detected upon BM-MSC-EV treatment of human peripheral blood mononuclear cells (PBMCs) (58, 59). In addition, the establishment of an anti-inflammatory and tolerant environment by BM-MSC-EVs is also favored by increased levels of IL-10, IL-6, transforming growth factor β (TGF-β), and prostaglandin E_2_ (PGE_2_) (48, 58, 60).

The effect of MSC-EVs has been investigated *in vitro* on B cells as well. In accordance with the observations on T cells, it has been demonstrated that BM-MSC-EVs are able to inhibit B-cell proliferation in a dose-dependent manner. Moreover, the treatment with these MSC-MVs affects the *in vitro* differentiation of human plasma cells from B lymphocytes, as well as the production of immunoglobulin (Ig) M, IgG, and IgA (63).

Despite these experimental findings on the immunomodulatory effect of MSC-EVs on adaptive lymphocytes, both the B cell–to–plasma cell ratio and the proliferation of T cells appear to be less affected by human MSC-EVs than by intact MSCs both *in vitro* and *in vivo*. These findings suggest that the cell–cell contact, although not essential, may play a pivotal role in the immunosuppressive potential of MSCs derived from UC, BM, and adipose tissue (51, 64, 65). Moreover, the immune regulatory effect of human BM-MSC-EVs could vary depending on the context and on the EV preparation. Thus, a careful investigation is essential to optimize their therapeutic potential (66).

#### MSC-EV Effect on Innate Immune Cells

In addition to the direct effect on adaptive immune cells, MSC-EVs also modulate innate immune responses. Furthermore, *in vitro* evidence demonstrates that BM-MSC-EVs can indirectly induce an immune-tolerant phenotype in T and B cells by inducing an anti-inflammatory state on APCs. Indeed, human peripheral blood (PB) granulocytes and monocytes are more prone to uptake BM-MSC-EVs than lymphocytes (67). To further support this observation, it has been shown that MSC secretion is not sufficient to promote Treg expansion, but the presence of additional mediators, including monocytes, is essential (68, 69). The stimulation with EXs isolated from human embryonic– or UC-derived MSCs induces an anti-inflammatory M2-like polarization in both human and murine monocytes, via the activation of TLR-dependent signaling. Such M2-like phenotype is characterized by an enhanced expression of anti-inflammatory IL-10 and TGF-β and an attenuated proinflammatory cytokine (IL-1β, IL-6, TNF-α, and IL-12P40) response. In turn, these M2 macrophages can promote a Treg phenotype in CD4^+^ T cells (53, 70, 71). A possible MSC-EV-mediated mechanism, determining this unbalance in favor of anti-inflammatory cytokines, could involve the cyclooxygenase 2 (COX2)–PGE_2_ axis. As a matter of fact, COX2 is contained in MSC-EVs, and its amount is particularly high in MSC-EVs preactivated with proinflammatory stimuli, as demonstrated by *in vitro* studies on EVs from human BM-isolated MSCs (67).

Similarly, the *in vivo* administration of human MSC-EXs increased the number of circulating Tregs in mice receiving a skin allograft, preventing graft rejection (53). Furthermore, *in vivo* tracking experiments in rats with damaged spinal cord demonstrated that BM-MSC-EXs localized into the injured site after infusion. This homing ability of MSC-EXs appeared to be mediated by macrophages, especially M2 (72). In agreement, a mouse model of renal dysfunction showed that BM-MSC-EXs can prevent the chemotaxis of activated macrophages into the inflamed organ, thus preventing the tissue damage caused by their accumulation (73).

Similar to monocytes, dendritic cells (DCs) can also be affected by MSC-EVs. *In vitro* studies in patients with type 1 diabetes demonstrated that human BM-MSC-EVs are able to induce an immature and resting phenotype in monocyte-derived DCs (moDCs), showing a reduced expression of CD80, CD86, CCR7, and HLA-II molecules. These moDCs produce high levels of IL-10, IL-6, TGF-β, and PGE_2_, thus potentially contributing to create an immune-suppressant microenvironment for T cells and leading to the induction of Treg during DC and naïve T-cell co-culture (74).

In addition to APCs, MSC-EVs can also modulate NK cell activity. In this regard, similarly to adaptive lymphocytes, *in vitro* studies demonstrated that human BM-MSC-EVs could suppress NK cell proliferation especially in presence of inflammatory stimuli (75). Moreover, the periocular injection of human MSC-EVs, in experimental rodent models of autoimmune type 1 diabetes and uveoretinitis, appeared to reduce the NK cell trafficking within the lesions (76, 77).

### EV Applications in GvHD

Growing evidence demonstrates that regulatory cells (Treg, NK cells, invariant NK T cells, multipotent adult progenitor cells, MSCs, myeloid-derived suppressor cells, innate lymphoid cells) could play a role in reducing GvHD incidence and severity. Thus, these cells have been tested as GvHD prophylaxis or therapy in clinical trials (78). Given their immunomodulatory effect, regulatory cell–derived EVs have been proposed as cell-free therapeutic tool to counterbalance the excessive activation of the immune system during GvHD.

In the clinical setting of HCT, BM-MSC-EXs have been safely infused for the treatment of a patient with steroid-refractory cutaneous and intestinal grade IV GvHD (50). The infusion of such EXs significantly ameliorated GvHD symptoms. These EXs carried anti-inflammatory molecules, including IL-10, TGF-β, and HLA-G, but not proinflammatory cytokines and apoptosis-inducing molecules (50). This case demonstrated the beneficial effect of MSC-EVs as anti-inflammatory and immune-modulatory mediators. The efficacy observed is probably due to a decline of proinflammatory cytokines (e.g., TNF-α, IL-1β, and IFN-γ) released by patient-derived PBMCs upon MSC-EV stimulation (50).

To better characterize the immunomodulatory properties of MSC-EVs, several murine models of GvHD have been used. In a mouse model of allo-HCT, the intravenous administration of UC-derived MSC-EVs significantly lowered the numbers of alloreactive T cells. Moreover, the serum levels of IL-2, TNF-α, and IFN-γ were reduced, whereas the IL-10 levels were increased. All these changes resulted in the reduction of the clinical manifestations of aGvHD, thus improving mice survival (51). Consistent with these findings, it has been recently reported that, in a mouse model of aGvHD, the systemic infusion of BM-MSC-EVs reduces the pathologic damage in multiple GvHD-targeted organs and prolongs animals' survival. This effect could be due to the ability of MSC-EVs to suppress the proliferation of CD4^+^ and CD8^+^ T cells and the differentiation of naive T cells to an effector phenotype, preserving naive Treg cells (79).

Bone marrow–derived MSC-EVs isolated from healthy donors are able to modulate the expression of CD45RA on CD4^+^ and CD8^+^ T cells from PBMCs *in vitro*, by determining a shift of effector (TE) and effector memory (TEM) T cell frequencies. In addition, MSC-EVs were able to promote IFN-γ production by CD4^+^ TE and TEM. All these effects appear to be mainly influenced by recipient responsiveness toward a certain MSC-EV preparation, thus suggesting that the *ex vivo* assessment of PBMC and MSC-EV interactions could predict *in vivo* anti-GvHD responses (66).

In addition to the effects of MSC-EVs in ameliorating aGvHD symptoms, EVs have also been tested in cGvHD. In a model of human-into-mouse xenogeneic cGvHD, it has been observed that CD73^+^ EXs derived from BM-MSCs can inhibit T_H_1 cell effector functions through the conversion of ATP to adenosine, thus modulating GvHD (80). Moreover, a reduction of CD4^+^ T-cell activation and lung infiltration, as well as the inhibition of T_H_17 pathogenic cells and the induction of Treg cells, was also observed. These effects resulted in a significant reduction of skin, lung, and liver fibrosis and a prolonged mice survival (52).

Taken together, these findings strongly suggest that BM-MSC-EVs could recapitulate the therapeutic efficacy of BM-MSCs for the treatment of acute and cGvHD.

Extracellular vesicles find application for GvHD treatment also as carrier of bioactive molecules, such as anti-miRNA oligonucleotides. These molecules, synthetically designed, can be passively or actively loaded into EVs and used to neutralize specific regulatory miRNAs (81). This EVs have been tested in a mouse model of GvHD to reduce dysregulation of miR155, which is involved in the regulation of inflammation, as well as innate and adaptive immune responses (82). MiR155 up-regulation has been observed in immune cells and in EVs in specimens from patients with evidence of intestinal GvHD (83) and in rodent GvHD experimental models (82). It has been shown that the dysregulation of miR155 in mouse model drives T_H_1 proinflammatory T-cell phenotype (84). In this context, the infusion of EVs loaded with anti-miR155 in preclinical models reduced differentiation toward T_H_1, T_H_9, and T_H_17 cells and skewed differentiation toward T_H_2 and Treg cells, thus ameliorating the manifestations of GvHD and increasing mice survival (85).

An additional proposed application of miRNA-carried EVs is the use of EVs derived from a T-cell line overexpressing a miR146 mimic, which plays a regulatory role in inflammatory response in both mice and humans (86). MiR146 plays a major role also in endothelial inflammatory responses and activation (87), essential for the early phase of aGvHD onset, prior to its clinical presentation. In fact, preventive use of drugs, which protect and reduce endothelium activation, resulted in a decrease of frequency of GvHD in humans (88, 89). Thus, we can assume that the use of EVs enriched with miR146 mimic could potentially reduce endothelium activation affecting the incidence of aGvHD.

Circulating EVs and their miRNA and protein cargo could be useful not only as putative therapeutic tool, but also as biomarkers in HCT. Levels and composition of circulating EVs appear to be altered after HCT and before GvHD onset (90). A retrospective study demonstrated that the altered expression of CD146, CD31, and CD140a on EV surface correlated with risk of developing aGvHD (91). This correlation with GvHD onset has been confirmed in a prospective study for CD146 and CD31 (92). Furthermore, expression change of several miRNAs was also observed in serum EVs before GvHD onset. Representative examples are miR155, with miR100 and miR194b in EVs (92), and miR423, miR199, and miR93 in serum-derived EXs (93). Further studies are needed to define the reliability of such biomarkers. Nevertheless, all these findings strongly suggest the potential clinical application as biomarkers after HCT.

### MSC-EV Effect on Hematopoietic Stem Cells

Several evidence demonstrated that MSC-EVs could also modulate HSC fate. In particular, different studies performed in both human and mouse models have shown that EVs, either MVs or EXs derived from BM-MSCs, embryonic stem cells, and mature megakaryocytes promote the *ex vivo* expansion of CD34^+^ cord blood HSCs (CB-HSCs), cord blood-mononuclear cells, and BM-derived HSCs (42, 94–96). Additionally, when added to co-cultured HSCs and MSCs, human BM-MSC-MVs further improve the expansion of CB-HSCs, thus suggesting that they could represent a promising therapeutic tool to generate a great number of HSC for transplantation purposes (42).

In agreement, a recent work showed that human BM-MSC-EVs can up-regulate the JAK/STAT pathway and increase the levels of phospho-STAT5 in *in vitro*–cultured CD34^+^ cells, enriched from leukapheresis (97). The involvement of the JAK/STAT signaling pathway in CD34^+^ cell proliferation is important in several hematologic neoplasms, including myelodysplastic syndromes and acute myeloid leukemia (AML). In addition, it has been shown that this pathway plays a significant role in promoting cell survival (98). As shown in both humans and mice, MSC-EV treatment could also modify the gene expression profile of CD34^+^ cells and favor survival directly or indirectly, through microRNAs and Piwi-interacting RNAs (96, 97, 99). Gene expression profile of CD34^+^ cells is also modulated by human MSC-EV–derived miRNAs through repression of the Wnt/β-catenin signaling pathway (42). Furthermore, both murine and human BM-MSC-EVs showed anti-apoptotic effect on CD34^+^ cells (97, 99). When human CD34^+^ cells are co-cultured with human BM-MSC-EVs, there is an up-regulation of anti-apoptotic genes, such as BIRC2, BIRC3, and NFKB, a down-regulation of pro-apoptotic genes, including CASP3 and CASP6, and a decreased phosphorylation of H2AX. Further evidence supporting the importance of MSC-EVs in promoting HSC survival derive from studies demonstrating that the infusion of both murine and human MSC-EVs into lethally irradiated mice reduces the radiation damage to BM-HSCs, resulting in a long-term survival (99, 100). In particular, the use of EXs and MVs in combination was found to be superior to either MVs or EXs alone (77).

In addition to the ability of MSC-EVs to promote HSC survival and proliferation, BM-MSC-EVs appear to possess homing potential. Indeed, it has been observed that human BM-MSC-EVs can up-regulate CXCR4 expression in CD34^+^ HSCs, increasing their migration from the PB to the BM niche (96). Very recent findings supported this enhanced HSC migratory ability both *in vitro* and *in vivo* in the presence of human BM-MSC-EV stimulation, although the CXCR4 up-regulation was not confirmed (97).

Altogether, these data strongly suggest that MSC-EV treatment appears to positively contribute to HCT engraftment, favoring HSC survival, proliferation, and migration to the BM niche. Thus, BM-MSC-EVs combined with HSCs may contribute to the reconstitution of hematopoietic microenvironment and represent a new therapeutic option.

### EV Applications in Promoting GvL and in Preventing Disease Relapse

Therapeutic effects of allo-HCT are to a large extent mediated by GvL effects, through alloreactive donor-derived immune cells. Unfortunately, beneficial GvL effects are reduced by prophylaxis and treatment of GvHD. Therefore, ensuring good GvL effects preventing GvHD remains the “holy grail” of allo-HCT (101–103). Several strategies (such as the use of cytokines, the selective depletion of alloreactive T cells, regulatory immune cell infusions—in particular NK transfer and DC vaccination—and novel pharmacological agents, such as bortezomib and azacytidine) have been investigated to enhance, support, and preserve the antileukemia effects without aggravating GvHD (104). In this setting, EVs potentially find application to stimulate immune cells and promote antileukemia alloreactive responses.

The role played by NK cells in antileukemia activity has been extensively investigated. Natural killer lymphocytes are an integral component of the innate immune system and represent important effector cells in cancer immunotherapy, particularly in the control of hematological malignancies (105). Natural killer–derived EVs (NK-EVs), purified from either cell culture supernatants or plasma of healthy volunteers, have been shown to lyse target human tumor cells *in vitro* (106) and show promising anti-tumor effects in preclinical studies without impacting normal cells (107). Natural killer–derived EVs contain cytolytic and cytotoxic proteins, such as perforin, granzymes A and B, granulysin, and Fas ligand (108, 109) able to kill malignant hematologic cell lines (107). However, the underlying mechanisms of specific killing of tumor cells mediated by NK-EVs remain unclear.

In addition to NK-EVs, the anti-tumor effect of DC-derived EVs (DC-EVs) for immunotherapy of cancer is under investigation in clinical trials (110). DCs are professional APCs which present antigen material to T lymphocytes activating an antigen-specific T-lymphocyte immune response. Anti-tumor DC-based vaccines have revealed their high efficiency in various murine tumor models (111, 112) and human xenografts in immunodeficient mice (113).

Dendritic cell–derived EVs carry all the functionally active molecules needed for the activation and the induction of anti-tumor T-cell immune responses (complexes of MHC class I and II with tumor antigens, as well as co-stimulatory and adhesion molecules such as CD80, CD86, and CD40) (114) and can act alone as cell-free anti-tumor vaccines. To efficiently activate anti-tumor immune responses by DC-EVs, the proper choices of tumor antigens to load EV-producing DCs and of factors stimulating the maturation of DCs are of great importance. Significant success in the treatment of tumors by DC-EVs has been achieved in murine models and in human cell lines. Other strategies using tumor-derived EVs to deliver antigens to DCs and stimulating GvL are under investigation (110).

Even though anti-tumor activity of NK- and DC-EVs has been demonstrated *in vitro* and in preclinical studies (105, 110), studies to stimulate GvL after allo-HCT are lacking.

Extracellular vesicles could also be used as biomarkers to monitor disease persistence or promptly detect early signs of relapse before and after HCT. In this context, higher levels of EVs in patients' sera compared to healthy donors are detected in many hematological malignancies (115–118). Moreover, changes in absolute EV counts and EV protein contents have been observed after induction chemotherapy and corresponded to blast reduction in the BM (117, 119). Furthermore, EVs from malignant cells express abundant surface proteins unique to their cell of origin (120). For example, EVs derived from multiple myeloma cells overexpress, on their cell membrane, proteins such as CD147, CD38, and CD138 (115, 121–123). Disease progression has been correlated with an increase of CD147^+^ EVs, whereas CD138^+^ EVs have been associated with the disease phase. Similarly, circulating EVs derived from AML cells are enriched with cancer-derived proteins such as CD34, CD13, and CD117 (115, 124, 125).

In addition to surface membrane proteins, EV cargo (miRNAs and proteins) could give relevant information about drug resistance and disease relapse (119, 125). For instance, it has been observed that the presence of different forms of TGF-β1 propeptide, latency-associated peptide (LAP), and mature TGF-β1 in plasma EXs reflects the effects of chemotherapy and might be used as an indicator of AML relapse (117).

Higher levels of miRNAs, including let7a, miR9, miR99b, miR150, miR155, miR191, and miR223, have been found in AML cell–derived EXs, ranging from 2- to 40-fold enrichment compared with the levels in parent cells (126). MiR155, in particular, is significantly dysregulated in serum EVs in many hematologic malignancies (127), and its levels correlate with high white blood cell counts in AML patients.

Thus, the characterization by molecular and cytofluorimetric technique of EVs cargo may be useful to measure and monitor blast persistence before and after HCT, as well as potential predictor of drug resistance and disease relapse in patients in complete remission.

## Conclusions and Perspectives

The role of EVs in the context of HCT is rapidly growing in recent years. Because of their low immunogenicity, the effective use of MSC-EVs as treatment of inflammatory disease and their immune-modulating properties make EVs potential candidates for the treatment of post-allo-HCT complications (53, 58, 75, 128). Besides, their role as biomarkers for prognosis and disease progression has emerged. Many studies are now focusing on the characterization of their cargo and the identification of molecules responsible for their effects. In addition, in several hematological malignancies, one of the most promising future applications of EVs is their potential as non-invasive liquid biopsies, given that they appear to reflect the cell of origin.

Nevertheless, EVs need to be carefully characterized to thoroughly identify their composition to exploit them as therapeutic tools and as reliable biomarkers. The possibility of using EVs in clinical settings raises important technical issues on large-scale EV production and characterization methods.

Methodological issues remain to be resolved, and further studies are needed to better standardize isolation protocols. For instance, no single biomarker has yet been validated in independent patient cohorts to identify preclinical signs of HCT complications.

Altogether, the studies reported in this review show that EVs are potential biomarkers and promising drug delivery vectors in the setting of HCT-associated complications. The potential applications of EVs may eventually help in the early diagnosis and treatment of several HCT complications.

## Author Contributions

BB, DM, GL, and CD contributed to the initial conception and designed the manuscript. MC, LB, FC, MT, CD, and LG provided study materials and critically reviewed the manuscript. GL, CD, MC, DM, and BB wrote the manuscript. All authors gave the final approval to the manuscript.

### Conflict of Interest

The authors declare that the research was conducted in the absence of any commercial or financial relationships that could be construed as a potential conflict of interest.
